# A Method of Duplicating a Plate without Taking Impression or Bite

**Published:** 1904-09

**Authors:** Bessie B. Bennett

**Affiliations:** Baltimore, Md.


### A METHOD OF DUPLICATING A PLATE WITHOUT TAKING IMPRESSION OR BITE.

By Bessie B. Bennett, D. D. S., Baltimore, Md.

(Written for *The American Journal of Dental Science.*)

For many reasons it is often necessary to duplicate a plate, and if the expression and articulation are especially good, the operator is desirous to make as near as possible a fac-simile of the original.

This may be carried out to the very letter, and a virtual recast of the original denture constructed with no help from the patient but the old plate itself.

If the plate be broken, wax it together carefully—being especially careful that no wax be on the lingual and palatal portion as the case may be in lower or upper.

Now wet the plate, pour some thickly mixed plaster in it—thus forming your model. Allow this to become hard, and without attempting to remove the plate, invert the model (plate attached) just as though you were investing a warped model. Pour the upper portion of the plaster, close the flask tightly and place the bolts in position, just as though you were preparing to vulcanize.

Now select a vessel (tin, porcelain or enamel kitchen ware) deep enough to hold the flask and have about three inches of depth over. Fill the vessel with ordinary moulding sand, place the flask therein and place the pan on the back of a stove or over a slow gas flame, allowing the sand oo heat through gradually.

As the sand becomes warmed the heat may be increased. Keep this up until the sand bubbles—let it boil for several minutes.

The flask may now be opened and the old rubber found so soft that it may, with ease, be entirely removed, new rubber packed in its place and vulcanized as usual—thus gaining an exact reproduction of the old plate without many preliminaries.

This idea is not original with myself—but I am not able to give "Honor to whom honor is due," as the name of the supposed originator is not known to me.

